# Recent Advances of Nanomaterials-Based Molecularly Imprinted Electrochemical Sensors

**DOI:** 10.3390/nano12111913

**Published:** 2022-06-03

**Authors:** Xinning Dong, Congcong Zhang, Xin Du, Zhenguo Zhang

**Affiliations:** Shandong Provincial Key Laboratory of Animal Resistance Biology, Key Laboratory of Food Nutrition and Safety, College of Life Sciences, Shandong Normal University, Jinan 250014, China; 201913010337@stu.sdnu.edu.cn (X.D.); zhangcc1026@163.com (C.Z.)

**Keywords:** electrochemical sensor, nanomaterials, molecularly imprinted, polymer

## Abstract

Molecularly imprinted polymer (MIP) is illustrated as an analogue of a natural biological antibody-antigen system. MIP is an appropriate substrate for electrochemical sensors owing to its binding sites, which match the functional groups and spatial structure of the target analytes. However, the irregular shapes and slow electron transfer rate of MIP limit the sensitivity and conductivity of electrochemical sensors. Nanomaterials, famous for their prominent electron transfer capacity and specific surface area, are increasingly employed in modifications of MIP sensors. Staying ahead of traditional electrochemical sensors, nanomaterials-based MIP sensors represent excellent sensing and recognition capability. This review intends to illustrate their advances over the past five years. Current limitations and development prospects are also discussed.

## 1. Introduction

MIP is vividly illustrated as a system of artificial “locks” for “molecular keys”. In this idea, the “key” refers to the target analyte, and its well-matched “locks” are made of MIP. This analogue of a natural biological antibody–antigen system contributes to the selectivity of molecularly imprinted electrochemical sensors [1]. MIP can be utilized to coat the electrode surface to prepare MIP electrochemical sensors. There have been many methods exploited to prepare MIP [2], including layer-by-layer self-assembly, in situ chemical polymerization, sol-gel, and electro-polymerization. Electro-polymerization is an electrochemical method for preparation of conductive or nonconductive thin MIP film onto the transducer surface [3]. It is the generation technology with most potential for a multitude of reasons. First of all, the electrode surface can be coated by a uniform MIP membrane via just one step. Secondly, its thickness and shape can be regulated by parameters such as voltage and current, which provides sensor preparation with high reproducibility [4]. In addition, MIP sensors can operate in body fluid environments, allowing preparatory processes to take place in an aqueous solution [5].

**Figure 1:**
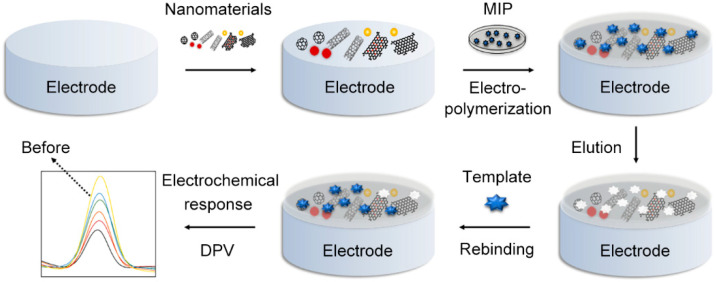
The construction and detection process of an MIP sensor.

The basic principle was illustrated in the schematic diagram of Figure 1. Firstly, nanomaterials were designed and synthesized, then modified on the polished electrode surface to enhance its conductivity and specific surface area. Secondly, MIP was electro-polymerized on it, and then template molecules were removed by solvent elution, leaving 3D cavities that could specifically match template molecules [6]. It was reported that the voltammetric sensors were the most common type; in this approach, electric signals would show a weakening trend after analytes’ recombination as the charge transfer between the electrode and redox probes was blocked. The content of analytes could be easily detected through quantitative analysis by linearizing the conversion values.

MIP sensors were designed for the detection of chemical compounds, proteins, cells [7,8], or other substances [9]. MIP-based sensors have many advantages. They can identify and recombine targets with high specificity, and are much cheaper than natural antibodies. Since they appeared, MIP sensors have been extensively applied in medical diagnosis [10,11], food safety [12,13,14], and environmental monitoring fields [10,15]. However, there are still some restrictions, such as uneven polymerization and low electric conductivity, which could be nicely solved by the attachment of nanomaterials [16].

## 2. Applications of Nanomaterials in MIP Sensors

Nanomaterials refer to materials with at least one dimension in nanoscale (0.1~100 nm) or that are composed of nanoscale units in three-dimensional (3D) space. Owing to their desirable properties, such as surface and interface effects, macroscopic quantum tunnel effect and quantum size effect, nanomaterials show great potential to improve the performance of overall electrochemical oxidation/reduction and alleviate the drawbacks concerning interference of other substances. In addition, sensors will be easily miniaturized and their adsorption capacity can be enhanced [17] with nanomaterials as the supporting materials [13].

### 2.1. Carbon Nanomaterials-Based MIP Sensors

The ratio of sp/sp^2^/sp^3^ hybridizations determines the formation of 3D carbon nanomaterials, 2D carbon sheets (graphene-based nanomaterials), 1D and hollow nanomaterials (carbon nanotubes), and 0D nanomaterials (carbon quantum dots) [18,19]. Besides the morphological structures, the ratio also decides their electrochemical properties [20].

#### 2.1.1. Carbon Nanotube-Based MIP Sensors

**Figure 2:**
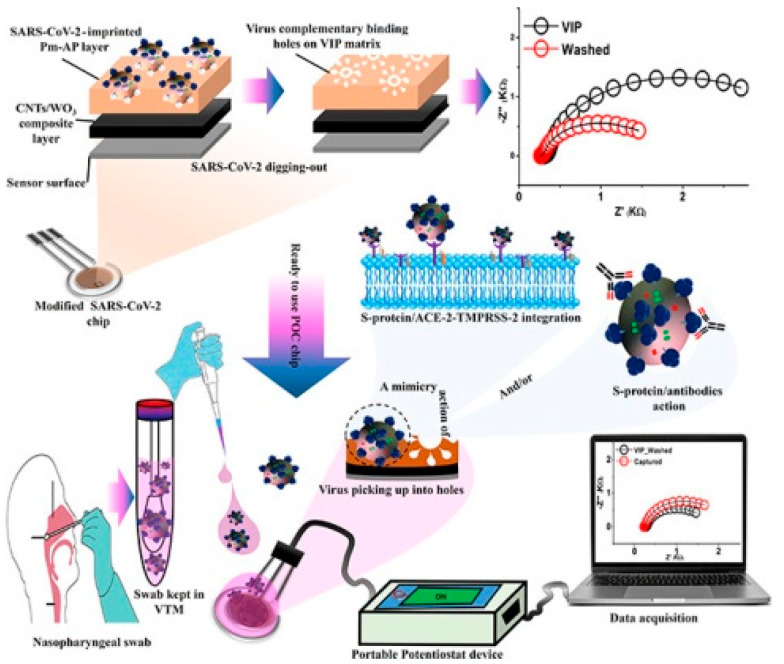
The construction and detection process of MIP sensors [24].

Formed by wrapped graphene, carbon nanotubes (CNTs) are mainly appearing as single-walled CNTs (SWCNTs) and multi-walled CNTs (MWCNTs). Having distinct thickness and metallic/semiconducting properties, both of them possess delicately adjustable electrochemical characteristics [21], such as rapid electron transfer capacity, large specific surface area, and easy functionalization [22,23]. Several studies demonstrated that CNTs could act as “electronic wire” between the surface of electrodes and the redox center of a protein, which significantly accelerated the direct electron-transfer reaction. Shumyantseva et al. [23] prepared a MWCNTs/MIP sensor via electro-polymerization of o-phenylenediamine (o-PD) and template myoglobin (Mb) on the MWCNTs’ modified screen-printed electrodes (SPE) surface. It was demonstrated to offer greater accuracy in undiluted plasma samples. Hussein et al. [24] developed a sensor by electro-polymerizing MIP on CNTs/WO_3_ modified SPE to detect SARS-CoV-2 particles. All preparation and detection processes were shown in the Figure 2. Its limit of detection (LOD) was 57 pg mL^−^^1^, and the electron transfer velocity was more rapid than that of other nanomaterial-based sensors.

Zhang et al. [16] polymerized a MIP film onto the vinyl group functionalized MWCNTs, which had better thermal stability. The hottest modification method was to treat MWCNTs with strong oxidants such as nitric acid to generate COOH. This carboxylated process could produce a large number of imprinted sites and oxygen-containing functional groups, which aided in adsorption, electron transport [20], and water dispersion of CNTs. Wu et al. [25] carboxylated MWCNTs with strong acids and then dropped them onto a polished glassy carbon electrode (GCE) surface to obtain MWCNTs/GCE. Subsequently, chitosan (CS) and tryptophan (Trp) mixture was applied to modify the GCE surface to form a MIP chitosan film. Characterized by cyclic voltammetry (CV), the oxidation current signal of MWCNTs/MIP/GCE was 2.1 times higher than that of MIP/GCE. Ma et al. [26] employed a composite of MWCNTs/CS and acrylamide (AAM) to a modified electrode. CS was not only used to provide amino groups for adsorption of glutaraldehyde (GA) and HIV-p24, but acted as a conductive bridge for electron transfer. Yu et al. [27] utilized MIP/SWCNTs-COOH/CS to modify GCE for semicarbazide (SEM) detection. Owing to the hydrogen bonding between CS and SWCNTs-COOH, the CS solubilization of CNTs was facilitated. Shaabani et al. [28] reported a sensitive sol-gel MIP sensor for urine naloxone (NLX) detection. NLX was added into initial sol and magnetically stirred for an hour to build the imprinted sol. After that, pyrrole (Py) was added to fabricate the Py@solgelMIP/fMWCNTs/ITO sensor. Because MIP and sol-gel were not electroconductive, nanomaterials were introduced. Carboxylated MWCNTs were evenly dispersed and made into a 3D reticular formation to increase current transport and binding sites. Akhoundian et al. [29] prepared a carbon nanocomposite paste electrode for ultra-trace trimipramine (TRI) analysis. MIP with a selective site was synthesized via precipitation polymerization by monomer vinyl benzene (VB) and methacrylic acid (MAA). The sensor could determine the concentration of TRI in a broad range, without pretreatment of serum samples and concerns about the interference effect caused by other substances.

The analytes, functional monomers, electrode modification, linear range, and LOD of CNT-based MIP sensors were listed in Table 1. Sensor properties are affected by the length and diameter of CNTs, so the modification processes should be fully characterized by a scanning electron microscope (SEM). There are still some implications that need to be taken into account, such as toxicity [30], biocompatibility, and the long-term effect on the ecosystem of CNTs [22].

#### 2.1.2. Graphene-Based MIP Sensors

In 2004, researchers discovered a method to prepare single-layer planar graphene sheets of atomic thickness [31]. Graphene is described as a large polycyclic aromatic with 2D sheets consisting of sp^2^ bonded carbon atoms. The surface area of graphene sheets is so large [32] that it offers numerous active sites for further modification [33]. Its superior conductivity was calculated as 64 mS cm^−^^1^, which was about sixty times stronger than that of SWCNTs. Liu et al. [34] developed a novel sensor by electro-polymerization of epigallocatechin-gallate (EGCG) and beta-cyclodextrins on electrodes with graphene oxide (GO) modification. This MIP sensor presented extraordinary advantages over conventional approaches because of the one-step preparation by CV scans without elution reagent. Liu et al. [17] successfully developed a GO-sheets modified MIP sensor for the detection of testosterone. The EIS response indicated the enhancement of sensitivity. Similar sensors could be further expanded to other endogenous substances. Several graphene-based MIP sensors were described in Table 2.

Moreira et al. [35] used reduced graphene oxide (rGO) to modify the GCE surface, and then electro-polymerized the MIP membrane by CV with phenol as the functional monomer. As a poor conductor, GO needed to be transformed to rGO [36], which contained fewer oxygen functional groups [37]. rGO@MIP preserved the merits of graphene@MIP and showed excellent conductivity and adsorption capacity [38]. The LOD of this sensor was seven times lower than that of high-performance liquid chromatography. Meanwhile, the morphological characterization revealed a rise in the surface roughness of rGO-based electrodes, which was conducive to the adhesion of MIP film and enhanced the imprinted cavities number up to 15 times. In their another research report, phenol was replaced with phenylboric acid (PBA) for selective detection of fructose [39]. PBA, which could form reversible borate esters through covalent interactions, was the suitable functional monomer for sugar recognition. Maryam Mostafavi et al. [40] synthesized sensors by rGO and polyaniline for the determination of diclofenac (DCF). It was shown via Fourier-transform infrared spectroscopy (FT-IR) and SEM imaging that the nano-layered structure of rGO made the electrolyte able to diffuse into the intervals of the sheets, thereby enhancing its conductive characteristics.

It was critical to make GO evenly distributed and fixed on the GCE surface [41], and dopamine (DA) had been discovered as an outstanding surface-adherent material. Bai et al. [42] fabricated an advanced MIP sensor by coating DA@graphene (DGr) and electro-polymerizing Py on the electrode. Polypyrrole (PPy) film fixed DGr. With the enhanced current response, the sensor could specifically recognize olaquindox (OLA) from its analogues, and its analysis of fish and feedstuffs showed a satisfactory result.

**Table 2:** Graphene-based molecularly imprinted sensors.

Analyte	Functional Monomers	Electrode Modification	Linear Range	LOD	Refs
EGCG	beta-CD	MIP/GO	3 × 10^−8^~1 × 10^−5^ M	8.78 × 10^−9^ M	[34]
Testosterone	o-PD	MIP/GO	1 × 10^−15^~1 × 10^−6^ M	4.0 × 10^−16^ M	[17]
D-xylose	Phenol	MIP/rGO	1.0 × 10^−13^~1.0 × 10^−10^ M	8.0 × 10^−14^ M	[35]
Fructose	PBA	MIP/rGO	1.0 × 10^−^^14^~1.0 × 10^−^^11^ M	1.1 × 10^−^^14^ M	[39]
DCF	Polyaniline	MIP/rGO	5~80 mg L^−1^	1.1 mg L^−1^	[40]
OLA	Py	MIP/DGr	5 × 10^−8^~5 × 10^−7^ M	7.5 × 10^−9^ M	[42]

#### 2.1.3. Carbon Quantum Dot-Based MIP Sensors

Carbon quantum dots (CQDs), consisting of carbon dots (CDs) and graphene quantum dots (GQDs), are 0D photoluminescent materials at the forefront of carbon nanomaterials research [43]. Derived from functionalized graphene, GQDs are several graphene sheets with lateral sizes below 100 nm. Owing to their outstanding electronic properties and low toxicity, CQDs have become suitable alternatives to conventional semiconductor QDs and carbon materials [44]. Although CQDs remain in the development stage, they are promising modifiers due to their simple synthesis and versatility. Applications of GQDs in MIP electrochemical sensors far exceed those of CDs [45,46].

Zheng et al. [47] proposed a novel MIP sensor with CDs and CS to improve its electron transportability. According to the study of Rao et al. [48], using Py as functional monomer, they electro-polymerized MIP film onto an electrode, which was modified by hollow nickel nanosphere-decorated GQDs. Based on the interaction between functional groups on GQDs and bisphenol S (BPS), functionalized GQDs could significantly improve the sensor detection velocity for BPS, which exhibited a LOD of 3 × 10^−8^ M under optimized conditions. Yao et al. [49] fabricated an electroconductive layer that was made of AgNPs and three-doped CQDs (B, N, F-CQDs). The sensitivity of the electrode was markedly improved by the synergy between them, which made it relevant for constant testing instead of chromatographic analysis. A PPy MIP membrane was further modified on the electrode. With a LOD of 1.12 × 10^−8^ M, the sensor could realize trace-level survey of analyte in plastic product samples.

Based on their unique fluorescence property, CQD-based fluorescence MIP sensors are an inspiring method. Jalili et al. [50] constructed a ratiometric fluorescent sensor with CDs as fluorophores. In this method, blue emissive CDs (B-CDs) were incorporated into silica-spheres, and yellow-emissive CDs (Y-CDs) were embedded in the MIP. When the 3D cavities on MIP film recombined with the analyte, the fluorescence color changed from the yellow to blue due to the fluorescence quenching of Y-CDs. The resulting ratios of fluorescence intensities at 560 nm and 440 nm could be used for trace level detection of penicillin-G in milk samples. Traditional QDs were generally extracted from lead, cadmium, and silicon, etc. Therefore, they were poisonous and deleterious to the environment. CDs have no relation to heavy metals and have excellent biocompatibility [51]. In practical applications, the main challenge is to seek innovative synthesis methods for high-quality CQDs with considerable yield [44].

### 2.2. Metal Nanoparticles-Based MIP Sensors

#### 2.2.1. Gold Nanoparticles-Based MIP Sensors

Inert metal nanoparticles have been broadly used to assess biological analytes, as their integration of aptamers and MIP can greatly improve the electrochemical performance of sensors [52,53]. Gold nanoparticles (AuNPs), with high biocompatibility, are essential to the sensor surface functionalization. They also provide the possibility of rapid electron transport between the electrode and the redox probe to enhance the resistance value changes. Zhang et al. [54] modified a gold electrode (GE) with AuNPs, polythionine-methylene blue (PTH-MB), and MIP in sequential order. Its current response was three times stronger than that of unmodified GE. The biosensor showed two oxidative peaks at −0.22 V and 0.20 V referred to PTH-MB and [Fe(CN)_6_]^3^^−^/^4^^−^, thus the amount of human serum albumin (HSA) in urine could be quantified by calculating the totality of double signals. In the investigation of Sehit [55], MIP/AuNP sensors had good specificity for glucose in human serum, whereas other sensors without AuNPs could not detect even the highest concentration of the research range. Yu et al. [56] constructed a MIP film on the electrode surface after AuNPs modification, which was generated by electro-polymerization of DA, polythymine aptamers, and melamine (MEL).

Motia et al. [57] functionalized the Au-SPE with AuNPs and electro-polymerized acrylamide/methylenebisacrylamide (AAM/NNMBA) on it to design an electrochemical MIP sensor for glycerol determination. The electro-kinetic analysis illustrated that the blend of AuNPs and polymer complex had a better sensitivity. In 2021, they [58] prepared another sensor for butylated hydroxyanisole (BHA) recognition, through electro-polymerization of AuNPs and CS. To demonstrate the role of AuNPs, they contrasted the sensitivity of sensors before and after modification, and then concluded that the sensitivity of (MIP-AuNPs)/SPCE was 12 times higher.

Recently, AuNPs have been incorporated into the modification of electrodes, acting as supporting materials for MIP. To obtain a high ratio of imprinted sites, AuNPs can be made into novel shapes with a larger surface area, and considering their good biocompatibility, they have already been applied in determination of organic and inorganic small molecules, such as protein or alcohol. Several AuNPs -based MIP sensors were listed in Table 3.

#### 2.2.2. Silver Nanoparticles-Based MIP Sensors

Silver is emerging as the best conductor among metals. Many properties of silver nanoparticles (AgNPs) have been widely acknowledged, such as large specific surface area, excellent biocompatibility, antimicrobial characteristics, and catalytic performance [59]. However, their applications in MIP sensors are slightly limited because of poor oxidative stability [60].

**Figure 3:**
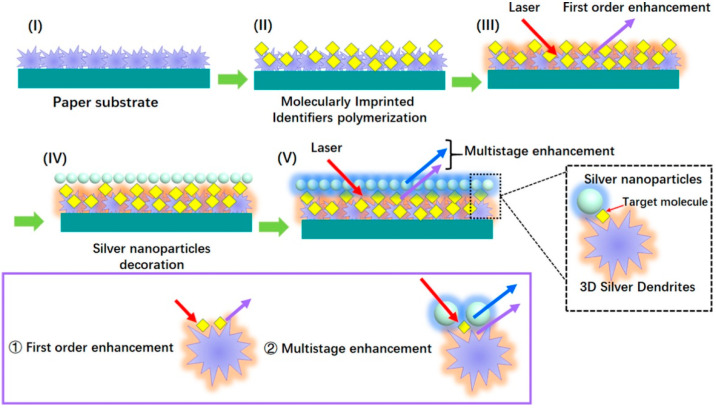
The 3D silver dendrites (SD)/MIP/AgNPs sensor was contributed through following steps [62]. (I) SD were grown on the paper substrate. (II) SD were coated by MIP film. (III) When laser light was incident, the first order enhancement generated but was not strong enough. (IV) The AgNPs layer was further decorated on it. (V) Multistage enhancement effect was produced by double Ag layers.

Nezhadali et al. [61] constructed a bi-layer of AgNPs and PPy for mebeverine (MEB). In this sensor, Py was electro-polymerized onto the electrode surface and then AgNPs were added by potentiostatic electrodeposition, which exhibited a brilliant electrocatalytic effect and amplified the electrochemical response. Noble metals had a surface plasmon resonance (SPR) effect and a AgNPs layer would have dense plasmonic hot spots to achieve ultrasensitive analysis. Zhao et al. [62] formed a sandwich structure of 3D silver dendrites (SD)/MIP/AgNPs by a paper-based surface-enhanced Raman scattering (SERS) amplified approach. SD in conjunction with AgNPs had constructed double Ag layers, and realized multistage enhancement of the SERS signal on the basis of the superposition of their electric and magnetic fields. The contribution processes of the sensor were illustrated in the Figure 3.

#### 2.2.3. Platinum Nanoparticles-Based MIP Sensors

Platinum nanoparticles (PtNPs) possess brilliant electrocatalytic activity and have been applied to MIP electrochemical sensing. For example, Xu et al. [63] firstly incorporated UiO-66 with PtNPs to detect phosalone (PAS). Using N-[3-(trimethoxysilyl) propyl] aniline (PAPTMS) and 3-aminobenzeneboronic acid (APBA) as functional monomer, active sites abounded on Pt-doped UiO-66 octahedrons. In tests of environmental samples, the sensors showed an ultra-low LOD (0.078 nM). Liu et al. [64] constructed a novel sensor by electrodepositing a dual-monomer MIP film on the electrode with Pt/Co_3_O_4_ nanoparticles modification. These transition metal oxides supported by noble-metal catalysts had a good catalytic activity and large surface area to enhance the detection sensitivity of the sensor. Under optimal conditions, this sensor exhibited a satisfactory anti-interference ability for chlorpromazine (CPZ) detection.

**Table 3:** Metal nanoparticles-based molecularly imprinted sensors.

Analyte	Functional Monomers	Electrode Modification	Linear Range	LOD	Refs
HSA	o-PD/HQ	MIP/AuNPs/ PTH-MB	1 × 10^−10^~1 × 10^−4^ g L^−1^	3 × 10^−11^ g L^−1^	[54]
Glucose	o-PD	MIP/AuNPs	1.25 × 10^−9^~2.56 × 10^−6^ M	1.25 × 10^−9^ M	[55]
MEL	DA/poly T	MIP/AuNPs	1 × 10^−12^~1 × 10^−4^ M	6.7 × 10^−13^ M	[56]
Glycerol	Acrylamide	MIP/AuNPs	20~227.81 μg mL^−1^	0.001 μg mL^−1^	[57]
BHA	CS	MIP/AuNPs	0.01 ~20 μg mL^−1^	0.001 μg mL^−1^	[58]
MEB	PPy	MIP/AgNPs	1 × 10^−8^~1 × 10^−6^, 1 × 10^−5^~1 × 10^−3^ M	8.6 × 10^−9^ M	[61]
IMI	EMIs	SD/MIP/AuNPs	0.2~800 ng mL^−1^	0.028 ng mL^−1^	[62]
PSA	PAPTMS/ APBA	MIP/PtNPs	5 × 10^−10^~2 × 10^−5^ M	7.8 × 10^−11^ M	[63]
Cocaine	PABA	MIP/PdNPs	1 × 10^−4^~5 × 10^−4^ M	5 × 10^−5^ M	[65]
CHO	PDA	MIP/Pt/AuNPs	1 × 10^−12^~5 × 10^−11^ M	2 × 10^−13^ M	[66]
6-MP	N-AAsp	MIP/N-HCNS@ PdNPs	0.8~70.748 ng mL^−1^	0.11~0.22 ng mL^−1^	[67]

#### 2.2.4. Palladium Nanoparticles-Based MIP Sensors

Palladium nanoparticles (PdNPs) are emerging noble metal nanomaterials, with outstanding electrical characteristics, oxidation resistance, and catalytic performance. Compared to other precious metal nanoparticles, such as gold, PdNPs are much cheaper and more easily available. Florea et al. [65] electrodeposited PdNPs on graphene-functionalized SPE (GPH-SPE). The deposition was conducive to the uniform distribution of p-aminobenzoic acid (PABA) and expanded the number of imprinted sites. In comparison with the single metal NPs, applications of bimetallic alloy NPs seemed to be much wider, and bimetallic Au/PdNPs were one of the most preferred alloys. Jalalvand et al. [66] electrodeposited Au/PdNPs and used polydopamine (PDA) as a functional monomer to establish a novel MIP sensor for cholestanol (CHO) test. Kumar et al. [67] decorated N-doped carbon nanospheres (N-CNS) with PdNPs and subsequently etched silica moieties to make them hollow (N-HCNS). N-acryloyl derivative of aspartic acid (N-AAsp), the appropriate functional monomer with carboxylic functional groups, has been applied to anchoring metal ions. Via electrostatic interactions, N-HCNS@Pd-MIP was adsorbed on the ionic liquid (IL) modified pencil graphite electrode. As a result of 6-mercaptopurine (6-MP) detection, the N-HCNS@Pd-MIP modified sensor obtained twice the heterogeneous rate constant, 3.5 times the diffusion coefficient, and 10 times electrical conductivity, compared to that of the N-CNS@Pd-MIP.

### 2.3. Metal Derivative Nanomaterials-Based MIP Sensors

#### 2.3.1. Metal Oxide Nanomaterials-Based MIP Sensors

Various metal oxide nanoparticles are composed of iron, zinc, copper, titanium, manganese, and so on [68]. Their unique properties, such as nontoxicity, piezoelectricity, and biocompatibility, have widened their applications in MIP electrochemical sensors [69]. For instance, Wang et al. [70] electrodeposited ZnO nanotubes (ZNTs) onto fluorine-doped tin oxide (FTO) glass to be high-surface 3D supports for MIP arrays. For electrodeposition of MIP, CV was performed in Py aqueous solution, and then in phosphate buffer solution to extract embedded DA. In this case, most imprinted sites were located at both internal and external surfaces of ZNTs, which shortened the charge transport pathway. Therefore, the sensor showed two dynamic linear ranges to DA, which were 0.02~5 μM and 10~800 μM. This preparation strategy could be further desired to be universal to imprint biomolecules. Yu et al. [71] added Py and nonylphenol into the TiO_2_ nanoparticles’ (TiO_2_NPs) dispersion. Then, NP-PPy@TiO_2_MIP suspension was dropped and dried on the cleaned GCE surface to form MIP. TiO_2_NPs, used as carriers, were capable of extending the detection range of p-nonylphenol (p-NP) to 1 × 10^−8^~8 × 10^−5^ M.

#### 2.3.2. Metal Sulfide Nanomaterials-Based MIP Sensors

Transition metal dichalcogenides (TMDs) are known as graphene analogues, which are made by stacking transition metal and sulfur sheets through van der Waals forces [72]. Molybdenum disulfide (MoS_2_) is a typical example.

MoS_2_ has attracted widespread attention in MIP fields such as hydrogen catalysis and storage because it shortens the electron transfer distance [73]. Significantly, with relatively low conductivity, it needs to accommodate additional nanomaterials to be operational [74]. Axin Liang et al. [75] modified GCE with highly conductive MoS_2_@N-GQDs-IL to magnify electrochemical signals for human immunoglobulin G (IgG). MoS_2_ was functionalized with nitrogen-doped GQDs (N-GQDs). Their integration with IL afforded an effective sensing platform, which contributed to the powerful electrostatic chemical interactions between MoS_2_@N-GQDs and IL. CuFe_2_O_4_ was used as the carrier to prepare the MIP. At the optimal conditions, the reaction was curtailed within 8 min with perfect accuracy and stability.

### 2.4. Nanocomposite-Based MIP Sensors

**Figure 4:**
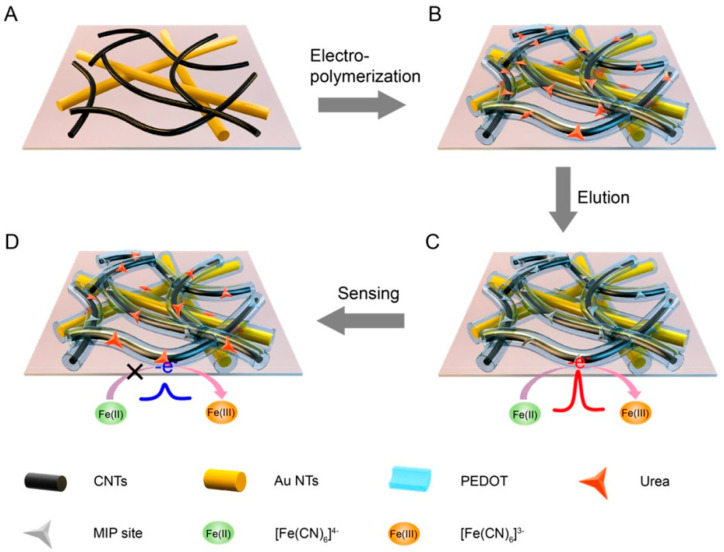
Fabrication of the MIP/C-AuNTs/PDMS EC sensor [77].

Nanocomposite, formed by nanomaterials combined with other organic or inorganic functional substances, is appropriate for functional utilization. Nanocomposite not only integrates the advantages of a single nanomaterial but also gains the synergistic effect of multiple components [53]. Wang et al. [76] modified AuNPs on cMWCNTs/GCE through chronoamperometry in HAuCl_4_. On this foundation, MIP film was electro-polymerized using o-PD as the monomer. This sensor finally had high sensitivity and could offer a 2.7 nM LOD for OLA. Liu et al. [77] designed a flexible electrochemical (EC) sensor with MIP and nanocomposite for urea detection in human sweat. As shown in the Figure 4, after the CNTs were loaded on AuNTs film, 3,4-ethylenedioxythiophene (EDOT) monomer was subsequently electro-polymerized. This strategy paved the way for epidermal noninvasive tests of other substances.

Chitosan (CS), with lots of amino groups and hydroxyl groups, is a biocompatible biopolymer with good film-forming ability and high mechanical strength [78]. Lian et al. [79] increased the electrochemical response through the synergistic effects of graphene-MWCNTs and CS-AgNPs. As the most important index of electrochemical sensors, it was essential to broaden the active area of the electrode surface and accelerate electrolyte diffusion. Guo et al. [80] modified the electrode with nanocomposite to acquire distinct electric signals. Homogeneously distributed CS-CDs and AuNPs ameliorated the bare GCE surface with more roughness. Moreover, introducing AuNPs for building the Au-S bond was an essential step to connect with the functional monomer ρ-Aminothiophenol (ρ-ATP). In comparison with conventional methods such as chromatographies and immunoassays, this sensor had higher sensitivity, faster processing speed, lower sample consumption, and lower cost.

rGO was usually compounded with AuNPs, and the nanocomposite had a faster electron transport speed and higher catalytic activity [81]. Beluomini et al. [82] electrodeposited rGO and AuNPs on GCE to develop a sensor for D-mannitol (D-M) determination in sugarcane vinasse. MIP was electro-polymerized on the AuNP/RGO-GCE by CV in acetate buffer solution containing D-M and o-PD. Its LOD was 7.7 × 10^−13^ M, and its selectivity to D-M was about six times as high as that of other reported electrochemical sensors. In a separate study, Zhang et al. [83] mixed the rGO with CS solution at a volume ratio of 1:1, and added the AuNPs for ultrasonic stirring to acquire AuNPs@rGO. Then, Py was electro-polymerized on AuNPs@rGO modified electrode. According to the report, this novel sensor could determine serum amyloid A (MAA) more rapidly, sensitively, and cheaply compared to the enzyme-linked immunosorbent assay (ELISA) method, which is the only present technique to monitor MAA. Yang et al. [4] developed a MIP-AuNPs-PDA-DGr/GCE sensor for ultra-trace monitoring of cholesterol (CHO). In this process, PDA was implemented not only to disperse graphene but also to produce a multifunctional platform on graphene to steady AuNPs and MIP films. Wrapped by PDA, AuNPs could grow into versatile Au microflowers. Au-S and intermolecular forces between PDA and functional monomers guaranteed the stable immobilization of MIP film. Wang et al. [84] successfully synthesized a polyethyleneimine (PEI)-rGO-gold nanoclusters (Au-NCs)@MIP sensor. Both rGO and AuNPs in nanocomposite could diminish the electrode surface resistance, making recognition of β-lactoglobulin (β-LG) faster and more accurate. Choline chloride (ChCl) and acrylic acid (AA) were used for hydrothermal synthesis of MIP. In actual samples tests, the LOD was 109 mg mL^−1^, much lower than the ELISA of 20 g mL^−1^.

We summarized attributes of several nanocomposites-based MIP sensors in Table 4. According to the characteristics of target analytes, it is promising to comprehensively use different nanomaterials with different functions and adopt various modification methods for preparing ideal sensors.

## 3. Conclusions and Prospects

Nanomaterials play an irreplaceable role in the advances of electrochemical sensors, which tremendously decrease their LODs to the femto level. In this review, we summed up the present advances of nanomaterials-based electrochemical MIP sensors. Most notably, nanocomposite is gradually becoming the mainstream in application of MIP electrochemical sensors, and portable, sensitive, and low-cost nanomaterials-based MIP electrochemical sensing devices will be more prevalent in the foreseeable future.

Further investigations of nanomaterials in MIP sensors are expected to be carried out focusing on three aspects. Firstly, according to the chemical structure of target analytes, it is significant to innovate new functional monomers with high specificity toward them. Secondly, we should pay more attention to the combination forms of nanomaterials and MIP in sensors to obtain a better overall performance. Last but not least, more effective fixation methods of modifiers in electrochemical MIP sensors are supposed to be exploited, as the leakage or exfoliation of modifiers can reduce the sensing surface sites and the electronic conductivity of the sensors.

## Figures and Tables

**Table 1:** CNT-based molecularly imprinted sensors.

Analyte	Functional Monomers	Electrode Modification	Linear Range	LOD	Refs
Mb	o-PD	MIP/MWCNTs	1 × 10^−^^11^~5 × 10^−^^10^, 5 × 10^−^^10^~5 × 10^−^^8^ M	1 × 10^−^^11^ M	[23]
SARS-CoV-2	m-AP	MIP/CNTs/WO_3_	7~320 pg mL^−1^	57 pg mL^−1^	[24]
Trp	CS	MIP/MWCNTs	2 × 10^−^^7^~1 × 10^−^^5^, 1 × 10^−^^5^~1 × 10^−^^4^ M	1 × 10^−^^9^ M	[25]
HIV-p24	AAM	MIPs/MWCNTs/CS	1 × 10^−^^4^~2 ng cm^−^^3^	0.083 pg cm^−^^3^	[26]
SEM	o-PD	MIP/SWNTs-COOH/CS	0.040~7.6 ng mL^−^^1^	0.025 ng mL^−^^1^	[27]
NLX	TEPS/Py	Py@solgelMIP/ fMWCNTs	0~1.2 × 10^−^^5^ M	2 × 10^−^^8^ M	[28]
TRI	MAA/VB	MIP/MWCNTs	1 × 10^−10^~2.5 × 10^−8^ M	4.52 × 10^−11^ M	[29]

**Table 4:** Nanocomposite-based molecularly imprinted sensors.

Analyte	Functional Monomers	Electrode Modification	Linear Range	LOD	Refs
OLA	o-PD	MIP/AuNPs/cMWCNTs	1 × 10^−8^~2 × 10^−7^ M	2.7 × 10^−9^ M	[76]
Urea	EDOT	MIP/C-AuNTs	1 × 10^−3^~0.1 M	1 × 10^−4^ M	[77]
Patulin	ρ-ATP	MIP/AuNPs/CS-CDs	1 × 10^−^^12^ ~1 × 10^−^^9^ M	7.57 × 10^−^^13^ M	[80]
D-M	o-PD	MIP/AuNPs/rGO	1 × 10^−12^~2 × 10^−11^, 2 × 10^−11^~3 × 10^−10^ M	7.7 × 10^−13^ M	[82]
MAA	Py	MIP/AuNPs@rGO	0.01~200 ng mL^−1^	5 pg mL^−1^	[83]
CHO	ATP	MIP/AuNPs/PDA/DGr	1 × 10^−^^18^~1 × 10^−^^13^ M	3.3 × 10^−19^ M	[4]
β-LAC	ChCl/AA	PEI-rGO-Au-NCs@MIP	1 × 10^−^^9^~1 × 10^−^^4^ mg mL^−1^	1 × 10^−^^9^ mg mL^−1^	[84]
